# Linear and nonlinear identification of the carotid sinus baroreflex in the very low‐frequency range

**DOI:** 10.14814/phy2.15392

**Published:** 2022-07-20

**Authors:** Toru Kawada, Tadayoshi Miyamoto, Ramakrishna Mukkamala, Keita Saku

**Affiliations:** ^1^ Department of Cardiovascular Dynamics National Cerebral and Cardiovascular Center Osaka Japan; ^2^ Department of Sport and Health Sciences, Faculty of Sport and Heath Sciences Osaka Sangyo University Osaka Japan; ^3^ Department of Bioengineering and Department of Anesthesiology and Perioperative Medicine University of Pittsburgh Pittsburgh Pennsylvania USA

**Keywords:** arterial baroreflex, Gaussian white noise, transfer function, Uryson model

## Abstract

Since the arterial baroreflex system is classified as an immediate control system, the focus has been on analyzing its dynamic characteristics in the frequency range between 0.01 and 1 Hz. Although the dynamic characteristics in the frequency range below 0.01 Hz are not expected to be large, actual experimental data are scant. The aim was to identify the dynamic characteristics of the carotid sinus baroreflex in the frequency range down to 0.001 Hz. The carotid sinus baroreceptor regions were isolated from the systemic circulation, and carotid sinus pressure (CSP) was changed every 10 s according to Gaussian white noise with a mean of 120 mmHg and standard deviation of 20 mmHg for 90 min in anesthetized Wistar‐Kyoto rats (*n* = 8). The dynamic gain of the linear transfer function relating CSP to arterial pressure (AP) at 0.001 Hz tended to be greater than that at 0.01 Hz (1.060 ± 0.197 vs. 0.625 ± 0.067, *p* = 0.080), suggesting that baroreflex control was largely maintained at 0.001 Hz. Regarding nonlinear analysis, a second‐order Uryson model predicted AP with a higher *R* ^2^ value (0.645 ± 0.053) than a linear model (*R* ^2^ = 0.543 ± 0.057, *p* = 0.025) or a second‐order Volterra model (*R* ^2^ = 0.589 ± 0.055, *p* = 0.045) in testing data. These pieces of information may be used to create baroreflex models that can add a component of autonomic control to a cardiovascular digital twin for predicting acute hemodynamic responses to treatments and tailoring individual treatment strategies.

## Graphical abstract

Although the arterial baroreflex is an important negative feedback system, its dynamic nature in the frequency range below 0.01 Hz remains to be elucidated. Despite the known resseting phenomenon to a conditioning pressure, the baroreflex maintained the dynamic gain when tested with a Gaussian white noise input with a switching interval of 10 s. These pieces of information may be used to create baroreflex models that can add a component of autonomic control to a cardiovascular digital twin for predicting acute hemodynamic responses to treatments and tailoring individual treatment strategies.

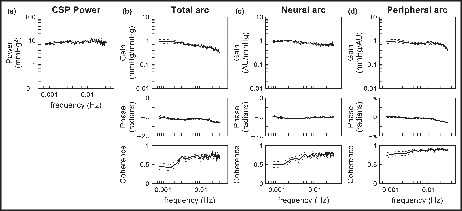

## 1 INTRODUCTION

The arterial baroreflex system is one of the major negative feedback systems to stabilize arterial pressure (AP) against pressure disturbances during daily activities. Since it acts immediately in response to pressure perturbations and yields full effect within a few minutes, the arterial baroreflex system is classified as an immediate control system (Hall, 2016). To identify the dynamic characteristics of the arterial baroreflex system, we have applied open‐loop analysis with the Gaussian white noise (GWN) approach (Kawada & Sugimachi, 2016). GWN inputs enable estimation of the linear and nonlinear dynamics of a given system even in the presence of significant noise contamination commonly encountered in physiological experiments (Marmarelis & Marmarelis, 1978). When tested in the frequency range between 0.01 and 1 Hz, the neural arc relating the carotid sinus pressure (CSP) input to the efferent sympathetic nerve activity (SNA) output reveals derivative characteristics (i.e., the magnitude response or dynamic gain increases with increasing frequency) (Ikeda et al., 1996). By contrast, the peripheral arc relating the SNA input to the AP output shows integral characteristics (i.e., the dynamic gain decreases with increasing frequency). It can be interpreted that the fast neural arc compensates for the slow peripheral arc to improve the rapidness of baroreflex‐mediated AP regulation (Ikeda et al., 1996). Nonlinear kernels of the total arc relating the CSP input to the AP output reveal an “Uryson” structure (i.e., products of the input only at the same time contribute to the output) and may enhance baroreflex buffering of AP increases in hypertensive conditions (Moslehpour et al., 2016a).

In our previous studies, the open‐loop transfer function of the baroreflex total arc approached a constant gain as the frequency decreased toward 0.01 Hz with an out‐of‐phase input–output relationship, suggesting that the baroreflex‐mediated AP response reached a near steady state at this frequency (Kawada & Sugimachi, 2016). Although the lowest frequency bound of 0.01 Hz allows the estimation of the baroreflex responses up to approximately 50 s (a half of the analyzed segment length), a prediction for a longer period may be required to simulate hemodynamic responses to drug administrations because some drugs take a few minutes to develop their effects. Whether the known dynamic characteristics of the arterial baroreflex can be simply extended down to 0.001 Hz remains to be examined because the baroreceptor transduction properties are known to reset toward a conditioning input pressure (Coleridge et al., 1981). If resetting occurs, the effective input magnitude may be reduced with time, resulting in an attenuated output. Hence, the dynamic gain of the baroreflex could be reduced in the frequency range below 0.01 Hz. On the contrary, pulsatile pressure does not induce significant baroreflex resetting compared to static pressure (Mendelowitz & Scher, 1990). It is also possible that the baroreflex can maintain the dynamic gain in the lower frequency range when an input pressure contains fluctuations of a wide frequency range such as in the case of the GWN input.

The aim of the present study was to estimate the dynamic characteristics of the carotid sinus baroreflex in the frequency range between 0.001 Hz and 0.01 Hz, a 10‐fold lower frequency range compared to our previous studies. First, we applied standard frequency‐domain transfer function analysis to elucidate the linear dynamics of this system. Second, we applied an extended nonparametric, frequency‐domain analysis to reveal the nonlinear dynamics of the system (Moslehpour et al., 2015, 2016a,b) with the expectation that the prolonged data sets might lead to new findings of baroreflex function.

## 2 MATERIALS AND METHODS

The experiments conformed to the Guiding Principles for the Care and Use of Animals in the Field of Physiological Sciences, which have been approved by the Physiological Society of Japan. The experimental protocol was reviewed and approved by the Animal Subjects Committee at the National Cerebral and Cardiovascular Center (No. 21009).

### 2.1 Surgical preparation

Eight male Wistar–Kyoto rats (326–435 g) were anesthetized with an intraperitoneal injection (2 mL/kg) of a mixture of urethane (250 mg/mL) and α‐chloralose (40 mg/mL). To maintain the anesthesia, the anesthetic mixture was diluted 18‐fold with physiological saline and administered continuously (2–3 mL·kg^−1^·h^−1^) through a catheter inserted into the right femoral vein. An arterial catheter was inserted into the right femoral artery for measuring AP. Ringer's lactate solution was administered (4 mL·kg^−1^·h^−1^) to maintain fluid balance. The rats were mechanically ventilated with oxygen‐enriched air, and the body temperature was maintained between 37°C and 38°C using a heating pad and a lamp.

After isolation of the bilateral carotid sinus baroreceptor regions from the systemic circulation (Sato et al., 1999; Shoukas et al., 1991), CSP was controlled using a servo‐pump system. Bilateral vagal and aortic depressor nerves were sectioned at the neck to minimize the reflex effects other than those mediated by the carotid sinus baroreflex.

A pair of stainless‐steel wire electrodes (AS633, Cooner Wire) were attached to a postganglionic branch of the left splanchnic sympathetic nerve and fixed with silicone glue (Kwik‐Sil, World Precision Instruments). The electrical signal was amplified, band‐pass filtered between 150 and 1000 Hz, full‐wave rectified, and then low‐pass filtered at a cut‐off frequency of 30 Hz to quantify SNA.

### 2.2 Protocol

CSP was changed every 10 s in steps according to a GWN signal with a mean of 120 mmHg and standard deviation of 20 mmHg. To avoid extreme input pressures from damaging the baroreceptors, the input pressures were limited between mean ± 3 standard deviations (i.e., 120 ± 60 mmHg).

After collecting the data during the application of the GWN input for more than 90 min, the static characteristics of the baroreflex were measured using a non‐pulsatile stepwise CSP input. CSP was decreased to 60 mmHg for 5 min and then increased to 180 mmHg in increments of 20 mmHg every minute.

At the end of the experiment, the animals were euthanized as follows. After inducing deep anesthesia with an additional intravenous administration of the above‐undiluted anesthetic mixture (2 mL/kg), the heart was arrested with an intravenous administration of a saturated potassium chloride solution.

### 2.3 Data analysis

The data were stored at 1000 Hz on a laboratory computer system via a 16‐bit analog‐to‐digital converter. 90‐min stationary portions of the data were analyzed. To identify the total arc, CSP and AP were treated as the input and output, respectively. To identify the neural arc, CSP and SNA were treated as the input and output, respectively. To identify the peripheral arc, SNA and AP were treated as the input and output, respectively.

### 2.4 Linear transfer function analysis

After resampling at 0.1 Hz (10‐s intervals), the input and output data were segmented into 7 half‐overlapping segments of 128 points each. The segment length was 1280 s, so the minimum investigated frequency (*f*_1_) was 0.00078 Hz. In each segment, the linear trend was subtracted, and a Hanning window was applied. The transfer function and magnitude‐squared coherence function were computed via standard nonparametric analysis per segment and then ensemble averaged (Bendat & Piersol, 2010; Kawada et al., 2019).

Since the amplitude of SNA varied considerably among animals due to different recording conditions, SNA was normalized in each animal and expressed in arbitrary units (AU) as follows. The minimum value of SNA obtained from the step input protocol was subtracted from the signal. Next, SNA was normalized per animal such that the average of the dynamic gain values of the neural arc transfer function below 0.003 Hz was unity. The inverse of the normalization factor was then applied to the peripheral arc transfer function.

### 2.5 Estimation of a second‐order Volterra model

For the nonlinear analysis, the 90‐min output signals were preprocessed to remove very slow trends. The mean values were subtracted, a second‐order Butterworth filter with a low‐cut frequency of 0.0005 Hz was applied in a bidirectional manner, and then the mean values were restored. After that, the 90‐min data were divided into a 60‐min segment for identifying the model and a 30‐min segment for testing the model.

The input–output relationship was assumed to be described by a second‐order Volterra series according to our previous studies (Moslehpour et al., 2015, 2016a,b) as follows:

(1) $$y\left(n\right)=h_{0}+\sum_{k=0}^{M}h_{1}\left(k\right)x\left(n-k\right)+\sum_{k_{1}=0}^{M}\sum_{k_{2}=0}^{M}h_{2}\left(k_{1},k_{2}\right)x\left(n-k_{1}\right)x\left(n-k_{2}\right)$$

where *x*(*n*) is the input signal after removing its mean value, and *y*(*n*) represents the output signal. The zeroth‐order kernel [*h*_0_] is the mean value of *y*(*n*). The first‐order kernel [*h*_1_(*n*)] describes how the present and past input samples affect the present output sample. The second‐order kernel [*h*_2_(*n*_1_, *n*_2_)] describes how the products of two present and past input samples affect the present output sample. The system memory (*M*) was set to 12 samples (or 120 s), which was significantly longer than the 25 s specified in our previous studies. The kernels were estimated via the previously reported, nonparametric, frequency‐domain method (Moslehpour et al., 2015, 2016a,b).

### 2.6 Estimation of a Uryson model

According to the results of our previous studies (Moslehpour et al., 2015, 2016a,b), we examined a second‐order Uryson model, which is a reduced form of the second‐order Volterra model, to describe the input–output relationship of the systems. The second‐order Uryson model is expressed as follows:

(2) $$y\left(n\right)=h_{0}+\sum_{k=0}^{M}h_{1}\left(k\right)x\left(n-k\right)+\sum_{k=0}^{M}h_{2U}\left(k\right)x^{2}\left(n-k\right)$$

where *h*_0_ and *h*_1_(*n*) are again the zeroth‐ and first‐order kernels, respectively, and *h*_2*U*_(*n*) is the second‐order kernel of the Uryson model. We also examined a third‐order Uryson model to describe the input–output relationship as follows:

(3) $$\begin{array}{c} y\left(n\right)=h_{0}+\sum_{k=0}^{M}h_{1}\left(k\right)x\left(n-k\right)+\sum_{k=0}^{M}h_{2U}\left(k\right)x^{2}\left(n-k\right)\\ \;+\sum_{k=0}^{M}h_{3U}\left(k\right)x^{3}\left(n-k\right) \end{array}$$

where *h*_3*U*_(*n*) is the third‐order kernel of the Uryson model. The kernels were analogously estimated via the previously reported, nonparametric, frequency‐domain method (Moslehpour et al., 2015, 2016a,b).

### 2.7 Prediction of the AP response to the stepwise CSP input

To understand how the estimated nonlinearity contributed to the prediction of AP, the AP response to the stepwise CSP input was predicted. The CSP input was changed from 60 to 180 mmHg in increments of 20 mmHg. The step duration of the simulation was set to 150 s considering the memory of 120 s. The steady‐state AP predictions were plotted against the CSP levels.

### 2.8 Statistical analysis

For the linear transfer function analysis, the gain and phase values were compared between 0.001 and 0.01 Hz using paired *t*‐tests. The average of two values at *f*_1_ and *f*_2_ (where *f*_*k*_ = *k* × *f*_*1*_) was used to represent the value near 0.001 Hz. The average of three values at *f*_12_, *f*_13_, and *f*_14_ was used to represent the value near 0.01 Hz.

For the nonlinear analysis, *R* ^2^ values between the measured and predicted outputs were compared among the linear model, Volterra model, and two Uryson models using paired *t*‐tests with the significance levels adjusted for _4_C_2_ = 6 comparisons via the Holm's method (Glantz, 2012). The *R* ^2^ values were log‐transformed before applying these tests for more normally distributed data (Bland & Altman, 1996).

## 3 RESULTS

**FIGURE 1:**
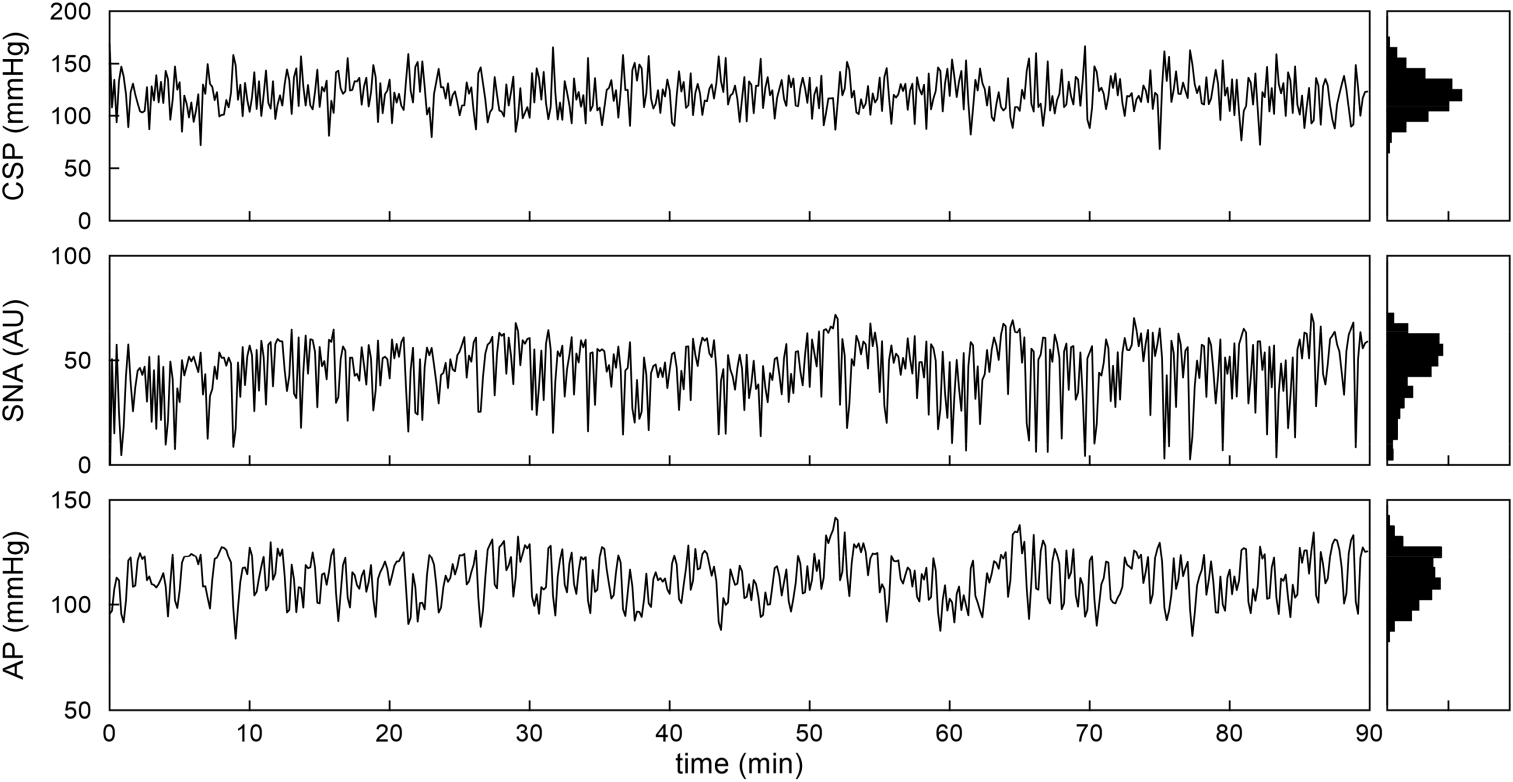
Typical recording of carotid sinus pressure (CSP), sympathetic nerve activity (SNA), and arterial pressure (AP) during a gaussian white noise CSP input with a mean of 120 mmHg and standard deviation of 20 mmHg. The input signal was changed every 10 s. In the histogram, CSP showed a Gaussian distribution, whereas SNA and AP did not. AU, arbitrary units.

Figure 1 shows typical time series obtained from one rat. CSP was perturbed according to a GWN signal. SNA and AP responded roughly reciprocal to the changes in CSP. As indicated by the histogram, CSP showed a quasi‐Gaussian distribution centered at 120 mmHg. The histograms for SNA and AP did not appear Gaussian, suggesting the presence of nonlinear SNA and AP responses.

### 3.1 Linear transfer function analysis

**FIGURE 2:**
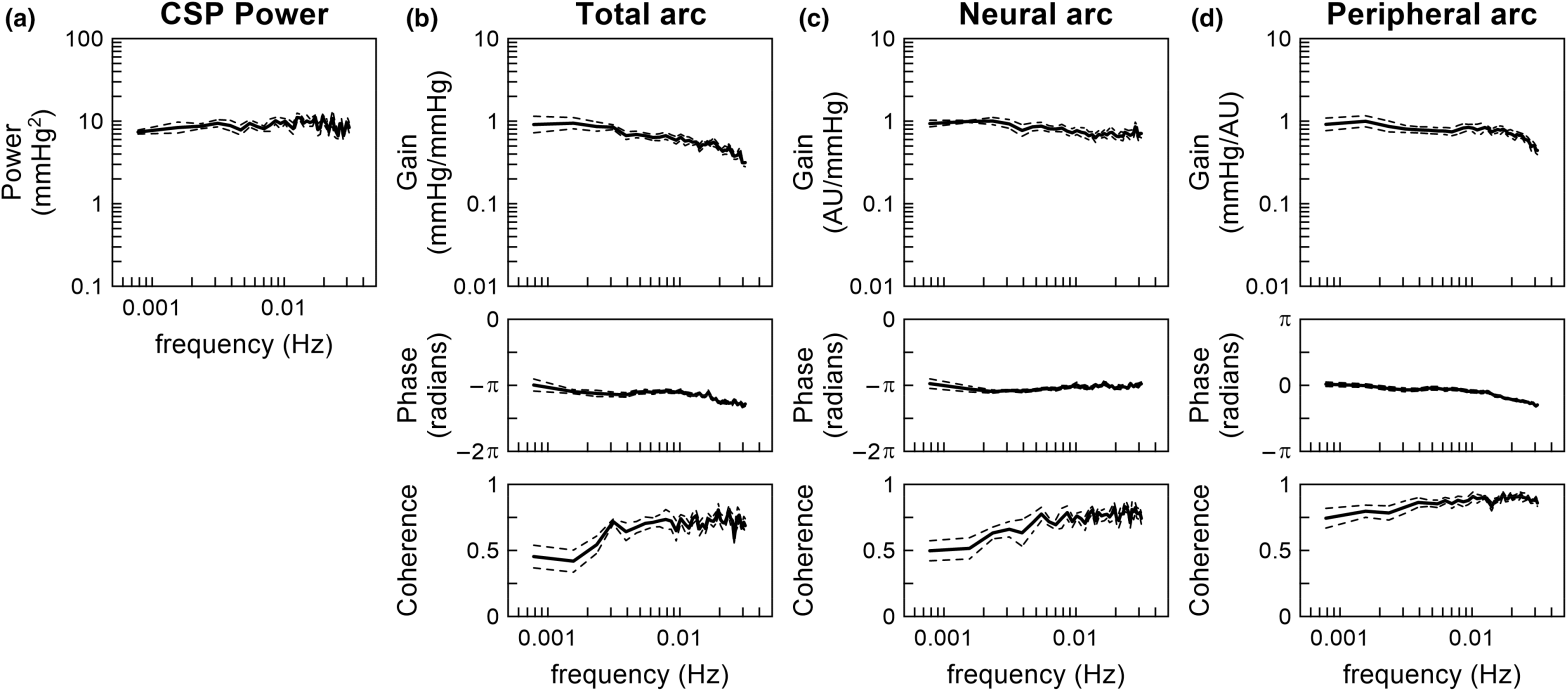
Pooled data of the carotid sinus pressure (CSP) input power (a), total arc transfer function (b), the neural arc transfer function (c), and peripheral arc transfer function (d). The lines indicate mean and mean ± SE values over eight rats. AU, arbitrary units.

Figure 2 illustrates the results of the linear transfer function analysis pooled over all eight rats. CSP had a relatively flat power spectral density in the frequency range between 0.00078 and 0.03 Hz (Figure 2a). For the total arc (Figure 2b), the dynamic gain tended to be higher at 0.001 Hz than at 0.01 Hz (Table 1). The slope of the dynamic gain between 0.001 and 0.01 Hz was −3.83 ± 1.73 dB/decade, which was significantly less negative compared to a first‐order system (*p* < 0.001 against −20 dB/decade by one‐sample *t*‐test). The mean value of the dynamic gain at 0.001 Hz was near unity, but this was not due to the normalization of SNA because the total arc relates CSP to AP. The phase was near −π radians at the lowest frequency, reflecting the negative feedback nature of the total arc. The phase values were not significantly different between 0.001 and 0.01 Hz (Table 1). The coherence was approximately 0.7 in the frequency range above 0.003 Hz and reduced as the frequency decreased toward 0.001 Hz.

**TABLE 1:** Gain and phase values of the linear transfer function

	0.001 Hz	0.01 Hz	*p* value
Total arc			
Gain, mmHg/mmHg	1.060 ± 0.197	0.625 ± 0.067	0.080
Log_10_(Gain)	−0.026 ± 0.081	−0.218 ± 0.040	0.062
Phase, radians	−3.23 ± 0.19	−3.46 ± 0.07	0.318
Neural arc			
Gain, AU/mmHg	0.981 ± 0.045	0.781 ± 0.096	0.124
Log_10_(Gain)	−0.011 ± 0.020	−0.127 ± 0.048	0.084
Phase, radians	−3.18 ± 0.18	−3.18 ± 0.09	0.987
Peripheral arc			
Gain, mmHg/AU	1.044 ± 0.153	0.852 ± 0.077	0.076
Log_10_(Gain)	−0.018 ± 0.070	−0.085 ± 0.047	0.115
Phase, radians	0.02 ± 0.09	−0.29 ± 0.07	0.038

Data are expressed as mean ± SE values (*n* = 8 rats). *p*‐values were determined by paired *t*‐tests.

For the neural arc (Figure 2c), the dynamic gain tended to be higher at 0.001 Hz than at 0.01 Hz (Table 1). The slope of the dynamic gain between 0.001 and 0.01 Hz was −2.31 ± 1.15 dB/decade. The phase values were close to −π radians, and did not differ significantly between 0.001 and 0.01 Hz (Table 1). The coherence was slightly higher than 0.7 in the frequency range above 0.004 Hz and reduced as the frequency decreased toward 0.001 Hz.

For the peripheral arc (Figure 2d), the dynamic gain tended to be higher at 0.001 Hz than at 0.01 Hz (Table 1). The slope of the dynamic gain between 0.001 and 0.01 Hz was −1.34 ± 0.74 dB/decade. In the present study, the mean value of the dynamic gain of the peripheral arc was near unity at 0.001 Hz when calculated using the normalized SNA values because the dynamic gain of the total arc was near unity at 0.001 Hz. The phase value was close to 0 radians at 0.001 Hz, and the phase of −0.29 ± 0.07 radians indicated a significant delay at 0.01 Hz (Table 1). The coherence was higher than 0.8 in the frequency range above 0.003 Hz and slightly reduced as the frequency decreased toward 0.001 Hz.

### 3.2 Nonlinear analysis

**FIGURE 3:**
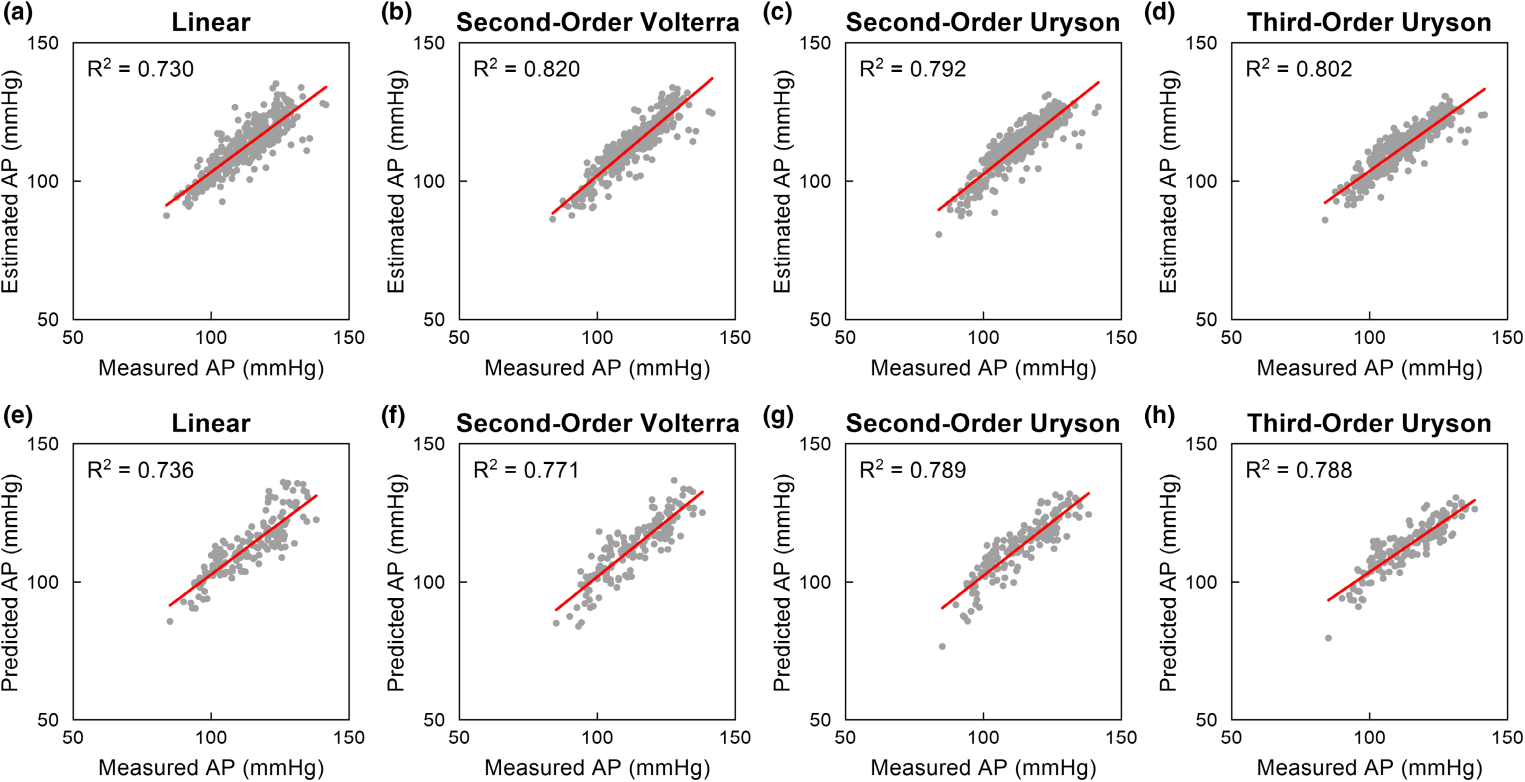
Scatter plots between measured and estimated arterial pressure (AP) values for a linear model (a), second‐order Volterra model (b), second‐order Uryson model (c), and third‐order Uryson model (d) obtained from the training data of one rat. Scatter plots between measured and predicted AP values for the linear model (e), second‐order Volterra model (f), second‐order Uryson model (g), and third‐order Uryson model (h) obtained from the testing data of the same rat. The red lines indicate the regression lines.

Figure 3 depicts typical results of the nonlinear analysis for the total arc obtained from one rat (the same rat as shown in Figure 1). Figure 3a–d represents scatterplots between the estimated and measured AP values obtained from the training data. The second‐order Volterra model showed the highest *R* ^2^ value among the four models. Figure 3e–h represent scatterplots between the predicted and measured AP values obtained from the testing data. The second‐order and third‐order Uryson models showed higher *R* ^2^ values than the linear and second‐order Volterra models.

Table 2 summarizes the *R* ^2^ values for the total arc pooled over all eight rats. In the training data, all three nonlinear models showed *R* ^2^ values higher than that of the linear model. The *R* ^2^ value was significantly higher for the second‐order Volterra model compared to the second‐order Uryson model, whereas there was no significant difference in the *R* ^2^ values between the second‐order Volterra model and the third‐order Uryson model. The *R* ^2^ values were not significantly different between the two Uryson models. In the testing data, the second‐order Volterra model did not show a higher *R* ^2^ value than the linear model. The *R* ^2^ values were higher for the two Uryson models than the linear model and the second‐order Volterra model. The *R* ^2^ values were not significantly different between the two Uryson models.

**TABLE 2:** *R* ^2^ values between measured and predicted outputs of the total arc

	Linear (L)	Second‐order Volterra (V)	Second‐order Uryson (U2)	Third‐order Uryson
Training data	0.610 ± 0.031	0.739 ± 0.025	0.706 ± 0.031	0.710 ± 0.032
*p*‐value vs. L		0.007†	0.046*	0.046*
*p*‐value vs. V			0.036*	0.092
*p*‐value vs. U2				0.407
Testing Data	0.543 ± 0.057	0.589 ± 0.055	0.645 ± 0.053	0.648 ± 0.055
*p*‐value vs. L		0.294	0.025*	0.026*
*p*‐value vs. V			0.045*	0.044*
*p*‐value vs. U2				0.793

Data are expressed as mean ± SE values (*n* = 8 rats). *p*‐values were determined by paired *t*‐tests of log‐transformed *R* ^2^ values with Holm's correction for multiple comparisons. Symbols * and † indicate *p* < 0.05 and *p* < 0.01, respectively.

**FIGURE 4:**
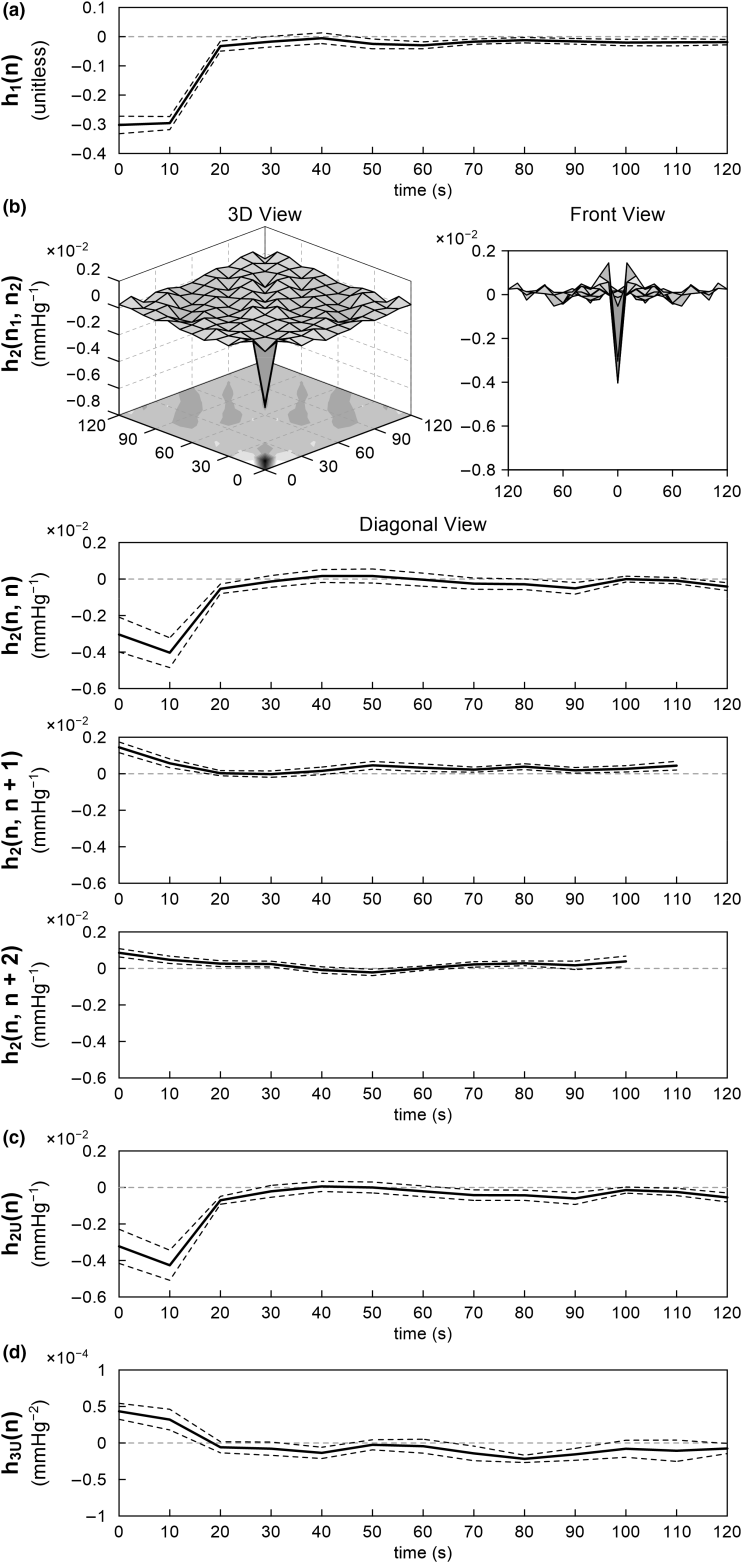
The pooled results of the nonlinear analysis of the total arc. (a) Linear kernel. (b) Second‐order Volterra kernel is illustrated in the 3D view, front view, diagonal view, adjacent off‐diagonal view, and next off‐diagonal view. (c) Second‐order Uryson kernel. (d) Third‐order Uryson kernel. The lines are mean and mean ± SE values over eight rats.

Figure 4 illustrates the estimated kernels for the total arc pooled over all eight rats. The linear kernel showed negative deflections at 0 and 10 s (Figure 4a). The kernel returned to near zero at 20 s. The second‐order Volterra kernel showed significant deflections only near the origin of both time axes (Figure 4b). In the diagonal view [*h*_2_(*n*, *n*)], corresponding to the system response to the squared input, the second‐order Volterra kernel showed a profile similar to the linear kernel, but the most negative value tended to occur at 10 s. In the adjacent off‐diagonal view [*h*_2_(*n*, *n* + 1)], corresponding to the system response to the product of the inputs one sample apart, the kernel showed a positive deflection at 0 s. In the next off‐diagonal view [*h*_2_(*n*, *n* + 2)], corresponding to the system response to the product of the inputs two samples apart, the positive deflection of the kernel became smaller at 0 s. The second‐order Uryson kernel resembled the diagonal view of the second‐order Volterra kernel (Figure 4c). The third‐order Uryson kernel showed positive deflections at 0 and 10 s (Figure 4d). The third‐order Uryson kernel showed some fluctuations beyond 70 s, but this could be due to the estimation error.

**FIGURE 5:**
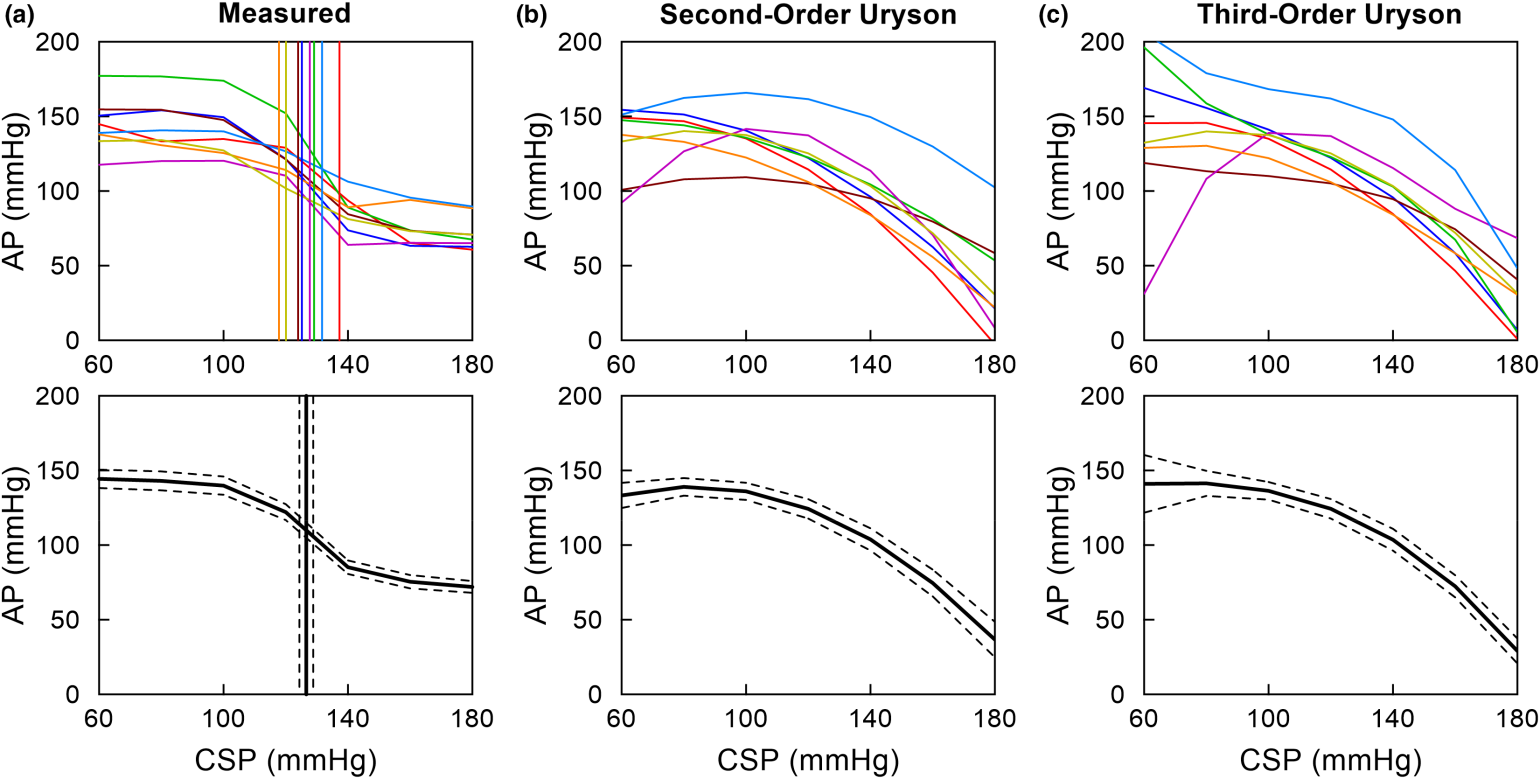
The relationship between carotid sinus pressure (CSP) and arterial pressure (AP) at a steady state during a stepwise CSP input. (a) Measured AP versus CSP. The vertical lines indicate the midpoint pressure of a four‐parameter logistic function fitted to the data points. (b) Predicted AP versus CSP for the second‐order Uryson model. (c) Predicted AP versus CSP for the third‐order Uryson model. In each panel, data derived from individual rats are shown in different colors in the top plot. Mean and mean ± SE values pooled over eight rats are shown in the bottom plot.

As shown in Figure 5a, the relationship between CSP and measured AP during the stepwise CSP input approximates an inverse sigmoid curve in individual rats (Figure 5a, top) and pooled over all eight rats (Figure 5a, bottom). This static relationship for the total arc showed the thresholding property around 100 mmHg, above which AP started to decrease with increasing CSP, and the saturation property around 140 mmHg, above which AP did not decrease further with increasing CSP. The vertical lines indicate the midpoint pressure of a four‐parameter logistic function fitted to the measured data. The second‐order and third‐order Uryson models showed a convex relationship between CSP and predicted steady‐state AP, capturing the thresholding property only (Figure 5b and c). The second‐order Volterra model, which did not improve the *R* ^2^ value over the linear model in the testing data, yielded inconsistent steady‐state AP predictions in response to the stepwise CSP input (not shown).

Table 3 summarizes the *R* ^2^ values for the neural arc pooled over all eight rats. In the training data, all three nonlinear models showed higher *R* ^2^ values compared to the linear model. There were no statistically significant differences in the *R* ^2^ values of the three nonlinear models. In the testing data, the two Uryson models, but not the second‐order Volterra model, showed higher *R* ^2^ values compared to the linear model. The *R* ^2^ values were not significantly different between the second‐order and third‐order Uryson models.

**TABLE 3:** *R* ^2^ values between measured and predicted outputs of the neural arc

	Linear (L)	Second‐order Volterra (V)	Second‐order Uryson (U2)	Third‐order Uryson
Training Data	0.719 ± 0.029	0.831 ± 0.015	0.817 ± 0.023	0.819 ± 0.026
*p*‐value vs. L		0.006 †	0.021 *	0.020 *
*p*‐value vs. V			0.662	0.761
*p*‐value vs. U2				0.661
Testing Data	0.667 ± 0.051	0.719 ± 0.033	0.782 ± 0.039	0.776 ± 0.039
*p*‐value vs. L		0.181	0.012 *	0.019 *
*p*‐value vs. V			0.024 *	0.006 †
*p*‐value vs. U2				0.633

Data are expressed as mean ± SE values (*n* = 8 rats). *p*‐values were determined by paired *t*‐tests of log‐transformed *R* ^2^ values with Holm's correction for multiple comparisons. Symbols * and † indicate *p* < 0.05 and *p* < 0.01, respectively.

**FIGURE 6:**
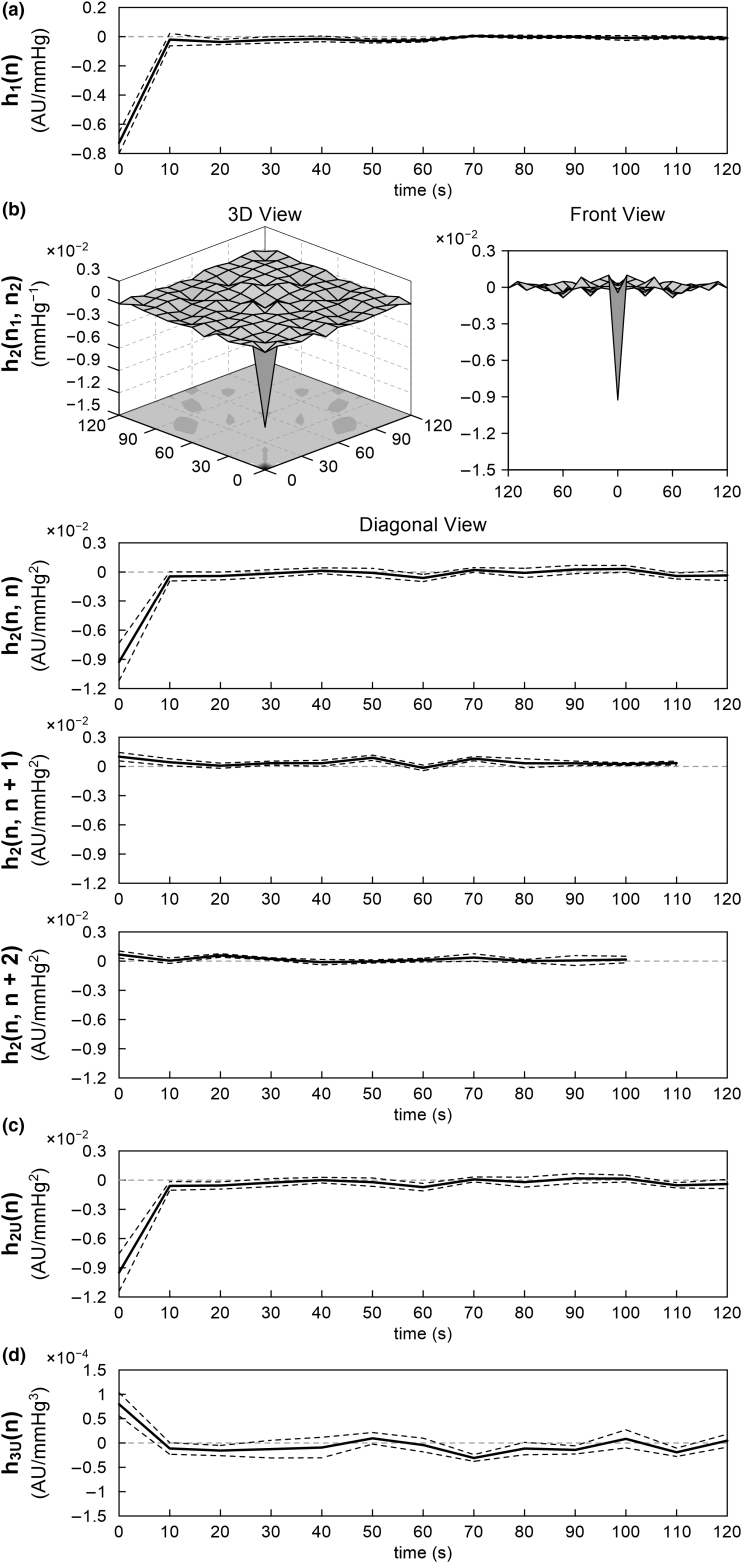
The pooled results of the nonlinear analysis of the neural arc. (a) Linear kernel. (b) Second‐order Volterra kernel is illustrated in the 3D view, front view, diagonal view, and adjacent off‐diagonal views. (c) Second‐order Uryson kernel. (d) Third‐order Uryson kernel. The lines are mean and mean ± SE values over eight rats.

Figure 6 illustrates the estimated kernels for the neural arc pooled over all eight rats. The linear kernel showed a negative deflection at 0 s (Figure 6a). The kernel returned immediately to near zero at 10 s. The second‐order Volterra kernel showed significant negative deflection only at the origin of both time axes (Figure 6b). In the diagonal view [*h*_2_(*n*, *n*)], the second‐order Volterra kernel showed a profile similar to the linear kernel. In the next off‐diagonal views [*h*_2_(*n*, *n* + 1) and *h*_2_(*n*, *n* + 2)], the kernel did not show significant deflections. The second‐order Uryson kernel resembled the diagonal view of the second‐order Volterra kernel (Figure 6c). The third‐order Uryson kernel showed a positive deflection at 0 s (Figure 6d).

**FIGURE 7:**
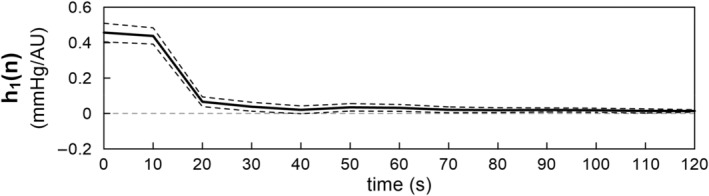
Linear kernel of the peripheral arc. The lines are mean and mean ± SE values over eight rats.

For the peripheral arc, the *R* ^2^ values derived from the linear model were 0.812 ± 0.030 and 0.805 ± 0.043 for the training and testing data, respectively. The nonlinear models did not yield better *R* ^2^ values compared to the linear model in the testing data (not shown). Figure 7 illustrates the estimated linear kernel for the peripheral arc pooled over all eight rats. The kernel showed positive deflections at 0 and 10 s. Thereafter, the kernel returned to zero.

## 4 DISCUSSION

We expanded our analysis on the dynamic characteristics of the arterial baroreflex system in the frequency range down to 0.001 Hz, as compared with 0.01 Hz in most of our previous studies (Kawada & Sugimachi, 2016; Moslehpour et al., 2015, 2016a,b). The major findings are that (1) the dynamic gain of the linear transfer function was maintained or tended to be greater at 0.001 Hz relative to that at 0.01 Hz (Table 1), (2) the nonlinear kernels did not show significant deflections beyond 20 s for the total arc (Figure 4b) and beyond 10 s for the neural arc (Figure 6b), (3) the second‐order Uryson model yielded better predictions compared to the linear model and second‐order Volterra model and as good predictions as the third‐order Uryson model for the total arc and neural arc in the testing data (Tables 2 and 3), (4) the Uryson models captured the thresholding property of the total arc (Figure 5b and c), and (5) the nonlinear models did not yield better predictions compared to the linear model for the peripheral arc.

### 4.1 Linear transfer function analysis

The baroreflex response to a static pressure input could be attenuated with time due to resetting of the baroreceptor transduction properties. One possible explanation is that connective tissues residing in series with the baroreceptor endings elongate with time and reduce the effective strain sensed by the baroreceptors (Coleridge et al., 1981; Mahdi et al., 2013). Ionic and chemical mechanisms may also be involved in baroreceptor resetting (Chapleau et al., 1989). Even in absence of the resetting of baroreceptors themselves, the conditioning pressure in the unilateral carotid sinus can cause baroreflex resetting in the contralateral carotid sinus by a central mechanism alone (Tan et al., 1989). The baroreceptor or baroreflex resetting can occur with a conditioning pressure as short as 20 min (Coleridge et al., 1981; Tan et al., 1989). In the present study, CSP was exposed to fluctuations over a wide frequency range (Figure 2a), but we did not observe substantial attenuation of the dynamic gain in the frequency range down to 0.00078 Hz, a frequency at which baroreceptor resetting could occur. As the input pressure continued changing every 10 s, baroreceptor resetting might have been prevented, as in the case of exposure to a pulsatile conditioning pressure (Coleridge et al., 1981).

In considering the buffering effects of the arterial baroreflex on AP variability for 24 h, Mannoji et al. modeled the dynamic characteristics of the total arc by a first‐order low‐pass filter with a pure delay where the corner frequency and the pure delay were set to 0.05 Hz and 0.85 s, respectively (Mannoji et al., 2019). The model was constructed based on the transfer function estimated in the frequency range from 0.01 Hz to 0.5 Hz and assumed no significant changes in the dynamic gain in the frequency range below 0.01 Hz. In the present study, the change in dynamic gain in the total arc between 0.001 and 0.01 Hz was small relative to a first‐order roll‐off (−20 dB/decade). Hence, no major improvements are proposed regarding the first‐order low‐pass filter with a corner frequency of 0.5 Hz to simulate AP regulation mediated by the arterial baroreflex. However, there is still room for argument that the total arc could show some dynamic characteristics in the frequency range far below 0.001 Hz.

### 4.2 Nonlinear analysis

The results of the nonlinear analysis for the total arc are basically consistent with our previous reports where the nonlinear kernels were estimated up to a memory length of 25 s from 10‐min data during the application of a GWN input with a switching interval of 0.5 s (Moslehpour et al., 2015, 2016a,b). In the present study, the second‐order Volterra kernel did not show significant deflections in memory length beyond 20 s, validating the presumptive memory length in the previous studies. The second‐order Volterra model provided a higher *R* ^2^ value than the second‐order Uryson model in the training data but not in the testing data (Table 2), suggesting overparameterization and less reliability of the Volterra kernel estimation. The third‐order Uryson model provided *R* ^2^ values similar to the second‐order Uryson model, indicating that the second‐order Uryson model sufficed to describe the dynamic characteristics of the total arc (Table 2).

As indicated in Figure 5a, the input–output relationship between CSP and measured AP at steady state approximates an inverse sigmoidal curve. Mathematically, second‐order nonlinear models can only capture either the thresholding or saturation property. In our previous studies and the present study, the second‐order Uryson model only captured the thresholding property (Figure 5b). While the third‐order Uryson model could capture both the thresholding and saturation properties, it captured only the thresholding property (Figure 5c) in agreement with our previous results (Moslehpour et al., 2016b). We have reasoned that the GWN input did not sufficiently excite the baroreceptors in the saturation zone. Indeed, the midpoint pressure of the measured sigmoid curve was 126.6 ± 2.2 mmHg (Figure 5a), whereas the mean of the GWN input was 120 mmHg, which made the number of input samples in the saturation zone less than that in the thresholding zone. Further studies are required to resolve the entanglement of the baroreflex static nonlinear properties.

For the neural arc, the linear and second‐order Volterra kernel showed a significant deflection only at 0 s, suggesting that the neural arc behaved as a purely static nonlinear system in the tested frequency range. Hence, from a neural arc perspective, identification in the frequency range as low as 0.01 Hz, which was done in our previous studies, suffices.

For the peripheral arc, the linear model yielded an *R* ^2^ value of approximately 0.81 in both the training and testing data, and the nonlinear models did not improve the *R* ^*2*^ value in the testing data, suggesting no significant nonlinear response of AP to the SNA input. However, it should be mentioned that the measured SNA was not GWN (Figure 1) and thus the nonlinear kernels of the peripheral arc may not have been well estimated. These results are consistent with our previous study using a shorter duration of data with CSP perturbation every 0.5 s (Moslehpour et al., 2015).

In this previous study, the linear model for the neural arc yielded an *R* ^2^ value of approximately 0.77 in the testing data, and the nonlinear models did not yield higher *R* ^2^ values compared to the linear model despite the known nonlinear properties of thresholding and saturation (Moslehpour et al., 2015). We were puzzled why the total arc showed significant nonlinearity whereas its subsystems, the neural and peripheral arcs, did not show significant nonlinearity. Measurement noise in SNA could have hampered the detection of the nonlinearity of the neural arc in our previous study. On the other hand, the linear model for the neural arc yielded an *R* ^2^ value of approximately 0.67 in the testing data in the present study, and the two Uryson models increased the *R* ^2^ values to approximately 0.78. As the data were processed every 10 s, the confounding effect of measurement noise could have been reduced, allowing the detection of the nonlinearity in the neural arc. In fact, the results for the total, neural, and peripheral arcs may be entirely consistent in the present study. The neural arc could be represented by a static system of the quadratic form (Figure 6), while the peripheral arc could be represented by a linear dynamic system of low‐pass characteristics (Figure 7), suggesting that the total arc could be represented by a Hammerstein system (i.e., a static nonlinear system followed by a linear dynamic system). The linear and second‐order Uryson kernels of the total arc were similar in shape and of low‐pass characteristics (Figure 4a and c). Assuming first‐ and second‐order kernels of the same shape, the total arc can indeed be reduced to a Hammerstein system for the 0.1 Hz resampled data (see Appendix). However, an Uryson model may be pertinent to describe the total arc over a wider frequency range up to 1 Hz.

### 4.3 Limitations

The number of animals was small (*n* = 8). The SE value of the dynamic gain at 0.001 Hz was three times greater than that at 0.01 Hz in the total arc. Increasing the number of animals could lead to statistical differences in the dynamic gain values between the two frequencies analyzed. However, the conclusion that the carotid sinus baroreflex largely retained the dynamic gain in the frequency range down to 0.001 Hz would still be the same. The vagi were cut bilaterally to remove vagal afferent signals and to achieve open‐loop conditions for the carotid sinus baroreflex. Accordingly, we could not assess the adaptation of the parasympathetic efferent arm from the heart rate response in the present data sets.

## 5 CONCLUSIONS

The dynamic characteristics of the carotid sinus baroreflex were examined in the frequency range down to 0.001 Hz. Linear analysis revealed that the dynamic gain at 0.001 Hz was maintained or tended to be greater compared with the dynamic gain at 0.01 Hz for the total arc, neural arc, and peripheral arc. Nonlinear analysis showed that second‐order Uryson models yielded better predictions compared to linear models for the total arc and neural arc. The linear model may however suffice to describe the dynamic characteristics of the peripheral arc. These pieces of information may be used to create baroreflex models that can add a component of autonomic control to a cardiovascular digital twin (Chakshu et al., 2021) for predicting acute hemodynamic responses to treatments and tailoring individual treatment strategies.

## AUTHOR CONTRIBUTIONS

T.K. and R.M. conceived and designed research; T.K. performed experiments and analyzed the data; T.K., T.M., R.M., and K.S. interpreted results of experiments; T.K. prepared figures and drafted manuscript; T.K. and R.M. edited and revised the manuscript; T.K., T.M., R.M., and K.S. approved the final version of the manuscript.

## CONFLICT OF INTEREST

The authors declare that there are no conflicts of interest.

## Figures and Tables

**FIGURE 8:**
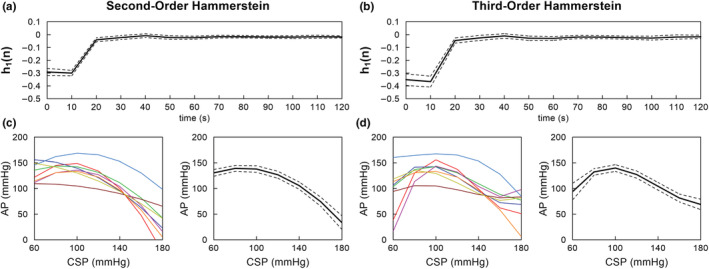
Linear kernels of the (a) second‐order and (b) third‐order Hammerstein models of the total arc. The predicted relationships between carotid sinus pressure (CSP) and arterial pressure (AP) at a steady state during a stepwise CSP input for the (c) second‐order and (d) third‐order Hammerstein models. In (c) and (d), data for individual rats are shown in different colors in the left plot. Mean and mean ± SE values pooled over eight rats are shown in the right plot.

## Appendix A

A Hammerstein system is comprised of a nonlinear static subsystem followed by a linear dynamic subsystem. When the nonlinear part is of polynomial type, the Hammerstein system can be represented by the following equation.

(A1)
$$y\left(n\right)=h_{0}+\sum_{k=0}^{M}h_{1}\left(k\right)\left[x\left(n-k\right)+b_{2}x^{2}\left(n-k\right)+b_{3}x^{3}\left(n-k\right)+\cdots \right]$$

where *b*_k_ is the coefficient for the *k*‐th polynomial term. The coefficient for the first‐order polynomial term (*b*_1_) can be fixed to unity so that *h*_1_(*n*) can be uniquely identified. We estimated the polynomial coefficients and the linear kernel for the total arc using a nonlinear least‐squares fitting method (Nelder & Mead, 1965). For a second‐order Hammerstein model, the initial value of *b*_2_ was set to 0.01 based on the relative magnitudes of the linear and second‐order Uryson kernels (Figure 4a and c). For the given *b*_2_, the linear kernel was estimated using standard nonparametric transfer function analysis, and the error between the estimated output and measured output was calculated. This process was repeated for values of *b*_2_ around the initial value, and the *b*_2_ value and linear kernel that minimized the sum of squared errors were selected as the final estimates. A third‐order Hammerstein model was likewise identified, starting with *b*_2_ and *b*_3_ set at 0.01 and 0.0001, respectively.

Table 4 shows the estimated polynomial coefficients. Figure 8a and b illustrate the linear kernels of the second‐order and third‐order Hammerstein models, respectively. Both models yielded *R* ^2^ values comparable to, but not better than, the second‐order Uryson model (Table 5). Although the third‐order Hammerstein model showed an *R* ^2^ value higher than the second‐order Hammerstein model in the training data, the *R* ^2^ values were not significantly different between the two Hammerstein models in the testing data. Figure 8c and d illustrate the prediction of the AP response to the stepwise CSP input derived from the second‐order and third‐order Hammerstein models, respectively. The Hammerstein models up to third‐order captured the thresholding property of the total arc. These results quantitatively confirm that the total arc can be represented by a second‐order Hammerstein model for 0.1 Hz resampled data.

**TABLE 4:** Polynomial coefficients for the second‐order and third‐order Hammerstein models

	*b*_2_ (×10^−2^)	*b*_3_ (×10^−4^)
Second‐order Hammerstein	1.605 ± 0.296	–
Third‐order Hammerstein	1.462 ± 0.385	−1.633 ± 0.752

Data are expressed as mean ± SE values (*n* = 8 rats).

**TABLE 5:** *R* ^2^ values between measured and predicted outputs of the total arc

	Linear (L)	Second‐order Uryson (U2)	Second‐order Hammerstein (H2)	Third‐order Hammerstein
Training data	0.610 ± 0.031	0.706 ± 0.031	0.695 ± 0.030	0.715 ± 0.029
*p*‐value vs. L		0.011 *	0.021 *	0.008 †
*p*‐value vs. U2			0.064	0.161
*p*‐value vs. H2				0.006 †
Testing data	0.543 ± 0.057	0.645 ± 0.053	0.654 ± 0.047	0.658 ± 0.048
*p*‐value vs. L		0.005 †	0.014 *	0.013 *
*p*‐value vs. U2			0.457	0.354
*p*‐value vs. H2				0.345

Data are expressed as mean ± SE values (*n* = 8 rats). *p*‐values were determined by paired *t*‐tests of log‐transformed *R* ^2^ values with Holm's correction for multiple comparisons. Symbols * and † indicate *p* < 0.05 and *p* < 0.01, respectively.
